# BMI, functional and cognitive status in a cohort of nonagenarians: results from the Mugello study

**DOI:** 10.1007/s41999-020-00417-9

**Published:** 2020-10-21

**Authors:** Monica Dinu, Barbara Colombini, Giuditta Pagliai, Federica Vannetti, Guido Pasquini, Raffaello Molino Lova, Francesca Cecchi, Sandro Sorbi, Francesco Sofi, Claudio Macchi, The Mugello Study Working Group, Roberta Boni, Chiara Castagnoli, Annamaria Gori, Anita Paperini, Lorenzo Razzolini, Nona Turcan, Debora Valecchi, Gianfranco Gensini, Rosanna Abbate

**Affiliations:** 1 Department of Experimental and Clinical Medicine, University of Florence, Largo Brambilla 3, 50134 Florence, Italy; 2 Don Carlo Gnocchi Foundation Florence, Onlus IRCCS, Florence, Italy

**Keywords:** Cognitive status, Functional status, Nonagenarians, Obesity paradox, BMI

## Abstract

**Purpose:**

The study of the relationship between body weight and health in old age has attracted increasing interest. The aim of the present study is to investigate the association of body mass index (BMI) with functional and cognitive status in a group of nonagenarians.

**Methods:**

We analyzed 475 participants (348 women, 127 men; median age 92 years) from the Mugello study. Participants were evaluated through laboratory, instrumental examinations and questionnaires.

**Results:**

By grouping the participants according to BMI categories, a better perception of health and nutritional status and a lower prevalence of sarcopenia (*p* < 0.05) were observed in participants with overweight and obesity compared to participants with normal weight or underweight. Concerning functional and cognitive measures, overweight and obese participants showed significantly worse performance on short physical performance battery and timed up and go tests and better performance on the mini-mental state examination (MMSE). As regards the other tests performed, no statistically significant differences were observed. In a multivariate logistic regression analysis adjusted for possible confounding factors, participants with BMI ≥ 30 kg/m^2^ showed lower probability to achieve poor performance on the MMSE (OR 0.42; 95% CI 0.19–0.94; *p* = 0.035).

**Conclusion:**

Our results support the hypothesis that in nonagenarians, a higher BMI is associated with better cognitive ability. Further studies are needed to explore the mechanisms underlying this association.

## Key summary points

**Aim:**

Our aim was to investigate the association of body mass index (BMI) with functional and cognitive status in a group of nonagenarians.

**Findings:**

By grouping the participants according to BMI categories, overweight and obese participants showed lower functional capability, higher risk of falling but better Mini-Mental State Examination (MMSE) performance than participants with normal weight or underweight.

**Message:**

This cross-sectional survey supports the hypothesis that adiposity could affect the cognitive state of people reaching the old age.

## Introduction

In recent years, many studies have linked functional and cognitive status to late life and body mass index (BMI) [1–3]. In terms of physical performance, a higher BMI has shown a controversial association with the effects on health and quality of life. Indeed, some studies have reported better physical functioning in older people with higher BMI [1], while others have reported more functional limitations and disabilities in those with higher BMI [4]. The same kind of results has been found in the literature between adiposity and cognitive state. An analysis of 2684 participants aged 65–94 [5] revealed a non-linear relationship between adiposity and cognitive function with overweight participants having better cognitive function, while Skinner et al. [6] reported significantly better cognitive flexibility performance in obese older adults.

The controversial association between adiposity and cognitive function has often been explained in the context of the “obesity paradox”, a hypothesis based on the fact that in old age obesity can have protective effects against certain conditions such as cognitive impairment [7]. The mechanisms underlying the “obesity paradox”, however, remain unclear, and not all the studies provide evidence to support this hypothesis [8, 9]. Moreover, there is little evidence on the relationship between BMI, functional and cognitive status in older populations.

The aim of this paper was to investigate the association of BMI with functional and cognitive status in old age, examining data from the Mugello study, a survey of people aged 90 years and older living in the Mugello area in Tuscany, Italy.

## Material and methods

### Study population

The analysis presented in this paper is based on data obtained from 475 nonagenarians (348 women, 127 men) enrolled within the Mugello study. The Mugello study is a cross-sectional survey of people aged 90 years and more, living in the Mugello area (north-east of Florence, Tuscany, Italy). The study protocol has been described in detail elsewhere [10]. Briefly, for each municipality of the Mugello area, nonagenarian residents and their general practitioners were contacted by letter and then by phone to ascertain their availability to participate in the study and to schedule a visit. The sample size (82% of all the nonagenarians living in the area) was highly representative of this population.

### Data collection

Participants were interviewed and examined at their home/nursing home by a trained physician, following a standardized protocol. General information on demographics, education, lifestyle, dietary habits, medical history, drug use and functional and cognitive status were collected from each participant. Level of physical activity was scored by administering a questionnaire modeled on the Harvard Alumni Questionnaire and adapted for the Italian population, as described elsewhere [10]. Weight and height were measured using a stadiometer. BMI was calculated as the weight (kg)/height (m^2^). Participants were classified as underweight if their BMI was < 18.5, normal weight if their BMI was 18.5–24.99, overweight if their BMI was 25–29.99, and obese if their BMI was 30 or more. Body composition was determined by a bioelectrical impedance analysis device (TANITA, model TBF-410).

The study was conducted in accordance with the principles of the Declaration of Helsinki on clinical research involving human beings and was approved by the Ethical Committee of the Don Gnocchi Foundation. Informed written consent was obtained from all participants, or their legal representative.

### Measures

Physical performance was evaluated using the Short Physical Performance Battery (SPPB) which assesses walking speed, standing balance and ability to raise from a chair. The total score ranges from 0 to 12, with higher score indicating better performance. Mobility was assessed by the timed up and go test (TUG). Patients were asked to rise from a 45 cm-high chair, walk forward 3 m at their usual walking pace, turn and walk back to the chair and sit down again. The score ranges from 0 to 3, with 0 indicating falls risk, 1 need assistance, 2 normal limits and 3 normal mobility. The maximal isometric handgrip strength (HGS, kg) was measured with a hydraulic dynamometer (RO + TEN, Verano Brianza, Italy). Participants repeated the test twice for each hand, and the best value for each side was recorded. The mean value of the best right and left results was used. Gait speed and functional mobility was evaluated through the Gait-speed test which measures time taken to walk 4 m. Gait speed of longer than 5 s to walk 4 m (≤ 0.8 m/s) suggests an increased risk of frailty. Performance of lower extremity muscles was measured using the sit-to-stand test. Participants were asked to stand up and sit down 5 times, as quickly as they could without any form of assistance. The time to complete the test has been recorded. Functional disability was assessed by the Basic Activities of Daily Living (BADLs) [11] and the Instrumental Activities of Daily Living (IADLs) [12] tools. Participants were classified as independent if they reported a score of 0–1 for all BADLs (eating, showering, dressing, transferring from bed to chair, using the toilet, and continence) and IADLs activities (shopping, doing light housework, preparing meals, managing money, using the telephone, taking medications, using transportation, and doing laundry), corresponding to perform all the considered activities without the help of another person.

The mini-mental state examination (MMSE) was used to tests 5 areas of cognitive function: orientation, registration, attention and calculation, recall and language [13]. The global score ranges from 0 to 30, with a score of 23 or less indicating severe (≤ 9 points), moderate (10–18 points) or mild (19–23) dementia. Depressive symptoms were investigated by the 15-item Geriatric Depression Scale (GDS) [14]. Scores of 0–4 were considered normal. Quality of life was assessed by the 12-Item Short-Form Health Survey (SF-12). Physical (PCS) and Mental (MCS) components were considered separately.

### Definition of sarcopenia

Sarcopenia was evaluated based on the diagnostic criteria of the European Working Group on Sarcopenia in Older People updated recommendations (EWGSOP2) [15]. Low muscle strength was defined as handgrip strength < 27 kg in men and < 16 kg in women. Low muscle quantity and quality was defined as appendicular skeletal muscle mass (ASM) < 20 kg in men and < 15 kg in women. Raw measures produced by bioelectrical impedance analysis were used for estimation of ASM mass, using the cross-validated Sergi equation for standardization [16]. Low physical performance was defined as SPPB ≤ 8-point score. Individuals with low muscle strength plus low muscle quantity and quality or low physical performance were categorized as sarcopenic.

### Statistical analysis

The statistical package PASW 20.0 for Macintosh (SPSS Inc., Chicago, IL, US) was utilized. Data were reported as mean ± standard deviation (SD), median and range or number and percentage, as appropriate. To analyze the possible relationship between BMI, functional and cognitive status, participants were grouped according to BMI categories. The Kruskal–Wallis test was used for comparisons among different groups. The Pearson’s Chi-square was used to test for proportions.

The association between BMI categories, functional and cognitive status was assessed using logistic regression analysis, with normal weight participants as reference group. Variables that were shown to be significantly different among groups in the descriptive analysis were introduced in a univariate model. A score ≤ 8 for SPPB, < 1 for TUG and ≤ 23 for MMSE was used as independent variable and BMI categories were used as dependent variables. A multivariate model was then performed to evaluate the associations after adjustment for possible confounding factors such as age, gender, marital status (widowed, married/partner, single/divorced), education (no diploma, elementary school, middle school, ≥ high school), smoking (yes, no), physical activity (more than once a week, no), waist-to-hip ratio (WHR), depression (GDS ≤ 4, > 4), sarcopenia (yes, no) and comorbidities (at least one of hypertension, diabetes, cardiovascular disease, and/or cerebrovascular disease, no). Results were reported as odds ratio (OR) and 95% confidence intervals (CI). Values were considered significant at *p* < 0.05.

## Results

The characteristics of the study population according to body weight status are reported in Table 1. Of the 475 participants, most were women (73.3%), and the median age was 92 years. Significant (*p* < 0.05) differences among BMI categories were observed for self-perception of general health and nutritional status, anthropometric parameters and prevalence of sarcopenia. Participants with overweight and obesity had better perception of their health and nutritional status, higher body fat mass, muscle mass and waist-hip ratio, and reported lower prevalence rates of sarcopenia compared with participants with lower BMI.

**Table 1:** Characteristics of study population according to BMI categories

	Underweight *n* = 27	Normal weight *n* = 227	Overweight *n* = 157	Obese *n* = 64	*p* value*
Age, years	93 (90–106)	92 (88–105)	92 (89–103)	91 (89–102)	0.311
Women, *n* (%)	24 (88.9)	167 (73.6)	108 (68.8)	49 (76.6)	0.149
Marital status					
Widowed,* n *(%)	20 (74.1)	164 (72.2)	126 (80.3)	55 (85.9)	0.310
Married/partner,* n* (%)	5 (18.5)	49 (21.6)	25 (15.9)	7 (10.9)	
Single/divorced,* n* (%)	2 (7.4)	14 (6.2)	6 (3.8)	2 (3.1)	
Living alone,* n* (%)	4 (14.8)	34 (15)	36 (22.9)	10 (15.6)	0.227
Education, years	3.9 ± 3.1	4.3 ± 3.1	4.4 ± 2.9	3.8 ± 2.2	0.206
Smoking status					
Current,* n* (%)	1 (3.7)	7 (3.1)	2 (1.3)	–	0.374
Former,* n* (%)	4 (14.8)	63 (27.8)	48 (30.6)	16 (25)	
Absent physical activity,* n* (%)	13 (48.1)	115 (50.7)	83 (52.9)	40 (62.5)	0.497
Health compared with peers^a^					
Better, *n* (%)	7 (25.9)	86 (37.9)	75 (47.8)	25 (39.1)	0.033
Worse, *n* (%)	3 (11.1)	22 (9.7)	7 (4.5)	4 (6.3)	
Good nutritional status^a^, *n* (%)	12 (44.4)	165 (72.7)	132 (84.6)	60 (93.8)	< 0.001
Anthropometric parameters					
Fat mass, %	11.2 ± 8.0	19.8 ± 9.7	27.8 ± 10.6	34.3 ± 10	< 0.001
Muscle mass, kg	23.7 ± 5.8	28.8 ± 8.5	31.1 ± 9.0	32.9 ± 6.6	< 0.001
Waist-hip ratio	0.93 ± 0.15	0.94 ± 0.09	0.95 ± 0.08	0.95 ± 0.07	0.031
Comorbidities and medications					
Cardiovascular diseases,* n* (%)	9 (33.3)	96 (42.3)	71 (45.2)	25 (39.1)	0.533
Cerebrovascular diseases,* n* (%)	4 (14.8)	41 (18.1)	36 (22.9)	15 (23.4)	0.453
Diabetes,* n* (%)	1 (3.7)	30 (13.2)	26 (16.6)	11 (17.2)	0.282
Hypertension,* n* (%)	11 (40.7)	96 (42.3)	82 (52.2)	34 (53.1)	0.162
Sarcopenia, *n* (%)	14 (51.9)	85 (37.4)	42 (26.8)	7 (10.9)	< 0.001
No of medications	3.3 ± 1.8	3.3 ± 2	3.8 ± 2	3.5 ± 2	0.133

Continuous variables are expressed as mean ± SD or as median (range). Categorical variables are expressed as number (percentage)

*p values calculated using Kruskal–Wallis test or *χ*^2^ test

^a^Self-perceived

Mean scores of physical and cognitive function tests according to body weight status are reported in Table 2. With regard to functional measures, significant differences among BMI categories were observed for SPPB (*p* = 0.005) and TUG (*p* = 0.010) tests. Participants classified as overweight and obese reported worse performance on both tests, denoting lower functional capability and higher risk of falling. With regard to cognitive tests, a significant difference among BMI categories was reported for MMSE (*p* = 0.003), with better performance in overweight and obese participants. No significant differences were observed for the other tests performed. By evaluating the functional independence, obese participants showed significantly (*p* = 0.004) lower functional independence (*n* = 14; 21.9%) compared with normal weight (*n* = 68; 30%) and underweight (*n* = 11; 40.7%) participants.

**Table 2:** Physical and cognitive function scores according to BMI categories

	Underweight	Normal weight	Overweight	Obese	*p* value*
*Physical function*					
SPPB (0–12 points)	4.2 ± 3.5	4.5 ± 2.9	3.9 ± 2.6	2.9 ± 2.2	0.005
TUG (0–3 points)	1.1 ± 1	1.5 ± 0.8	1.2 ± 0.8	1.1 ± 0.9	0.010
HGS test	11.6 ± 5.8	15.0 ± 7.5	14.9 ± 7.0	14.4 ± 5.8	0.155
Gait-speed test, m/s	0.5 ± 0.3	0.5 ± 0.3	0.4 ± 0.3	0.4 ± 0.2	0.315
Sit-to-stand test, s	24.4 ± 16.1	21.3 ± 13.1	21.7 ± 11.4	23.7 ± 15.0	0.752
BADLs (0–6 points)	3 ± 2.5	3.3 ± 2.3	3.5 ± 2	3.2 ± 1.9	0.700
IADLs (0–8 points)	3.2 ± 3.3	3.8 ± 3.4	3.9 ± 3.2	4 ± 3	0.668
PCS (0–100 points)	43.4 ± 8.6	43.6 ± 8.1	42.6 ± 7.9	41 ± 7	0.213
*Cognitive function*					
MMSE (0–30 points)	14.5 ± 9.7	18.4 ± 10	19.7 ± 8.8	21.9 ± 7.7	0.003
MCS (0–100 points)	45 ± 6.3	47.7 ± 7.5	47.4 ± 6.9	46.7 ± 7.1	0.644
GDS (0–15 points)	6.3 ± 4.6	4.4 ± 3.7	5.1 ± 3.9	4.2 ± 4	0.104

Data are expressed as mean ± SD

*SPPB* short physical performance battery, *TUG* timed up and go, *HGS* hand grip strength, *BADL* basic activities of daily living, *IADL* instrumental activities of daily living, *PCS* physical component summary scores, *MMSE* mini-mental state examination, *MCS* mental component summary scores, *GDS* geriatric depression score

*p values calculated using Kruskal–Wallis test

Finally, the relationship between BMI categories, physical and cognitive tests was assessed using logistic regression analysis. As reported in Table 3, participants with BMI ≥ 30 kg/m^2^ reported significantly higher risk of functional impairment (SPPB ≤ 8 points) (OR 4.47; 95% CI 1.30–15.33; *p* = 0.017) and falls (TUG < 1 point) (OR 2.58; 95% CI 1.15–5.79; *p* = 0.022), and lower probability to achieve poor performance on the MMSE (≤ 23 points) (OR 0.50; 95% CI 0.28–0.87; *p* = 0.015). After adjustment for possible confounding factors such as age, gender, marital status, education, smoking, physical activity, waist-hip ratio, depression, sarcopenia and comorbidities, the associations with functional impairment and falls risk lost their statistical significance. As regards the MMSE performance, the association remained statistically significant (OR 0.42; 95% CI 0.19–0.94; *p* = 0.035) in participants with BMI ≥ 30 kg/m^2^.

**Table 3:** Logistic regression analysis for the association between BMI categories, functional and cognitive tests

Variable	Univariate analysis			Multivariate analysis^a^		
	OR	95% CI	*p* value	OR	95% CI	*p* value
SPPB ≤ 8						
Underweight	0.04	0.27–4.00	0.955	1.94	0.28–13.36	0.500
Normal weight		Reference			Reference	
Overweight	1.76	0.93–3.35	0.085	1.55	0.71–3.37	0.274
Obese	4.47	1.30–15.33	0.017	3.43	0.89–13.20	0.073
TUG < 1						
Underweight	3.19	0.97–10.52	0.056	5.97	1.27–28.14	0.024
Normal weight		Reference			Reference	
Overweight	1.82	0.95–3.49	0.069	1.55	0.71–3.39	0.274
Obese	2.58	1.15–5.79	0.022	1.69	0.64–4.44	0.291
MMSE ≤ 23						
Underweight	1.37	0.59–3.21	0.706	0.80	0.26–2.52	0.555
Normal weight		Reference			Reference	
Overweight	0.93	0.61–1.40	0.710	1.18	0.67–2.08	0.572
Obese	0.50	0.28–0.87	0.015	0.42	0.19–0.94	0.035

*SPPB* short physical performance battery, *TUG* timed up and go, *MMSE* mini-mental state examination

^a^Adjusted for age, gender, marital status (widowed, married/partner, single/divorced), education (no diploma, elementary school, middle school, ≥ high school), smoking (yes, no), physical activity (more than once a week, no), waist-hip ratio, depression (GDS ≤ 4, > 4), sarcopenia (yes, no) and comorbidities (at least one of hypertension, diabetes, cardiovascular disease, and/or cerebrovascular disease, no)

## Discussion

The relationship between BMI and health in old age has been a controversial issue for many years. By grouping a large cohort of nonagenarians according to BMI categories, in the present paper we were able to report that overweight and obese participants had a lower functional capability, a higher risk of falling but better MMSE performance than participants with normal weight or underweight, thus supporting the hypothesis that adiposity could affect the cognitive state of people reaching the old age.

To date, the interaction between body weight, adiposity and cognitive function in the ninth decade of life is poorly understood [17]. Cognition is an extremely complex activity influenced by a multiplicity of biological cerebral mechanisms [18], and the comorbidities associated with overweight and obesity such as diabetes, cardiovascular disease, hypertension and sleep apnoea syndrome may explain most of the association between BMI and increased risk of cognitive impairment [19]. In older people, on the other hand, several studies suggested that the direction of the relationship between BMI and cognitive impairment may change in a counterintuitive way [20]. The negative effect of high BMI on cognitive functions in older people is less clear than in young adults and middle-aged people [21], and both positive and negative associations have been reported from studies on BMI in later life and dementia risk [6, 22].

In the present study, nonagenarians with a high BMI, without reporting a significant prevalence of sarcopenia and despite having a higher risk of falling and of functional disabilities, presented better MMSE scores. These results are in line with several papers reporting the so-called “obesity paradox” hypothesis [22, 23], that is a reduced risk of cognitive decline associated with overweight in late life, even if the issue remain controversial [24]. A very recent observational study investigating the relationship between obesity and cognitive impairment in 1100 patients aged 60–98 years has shown that overweight was associated with a lower risk of cognitive impairment [25], and the results of a prospective study revealed that older people with higher BMI had a lower risk of dementia than their counterparts with lower BMI [26]. On the other hand, other studies found no association between BMI and dementia [27], and several data showed an increased risk of cognitive impairment with a higher BMI in mid-life, reporting a negative effect of body weight gain on cognitive function in old age [21].

Therefore, the possible mechanisms linking adiposity and improved cognitive function in older age remain unclear, leaving room for speculation. One possible hypothesis is the “survival effect”. This hypothesis suggests that people who are susceptible to the negative effects of obesity die earlier, and those who survive into old age may be an obese subgroup resistant to such effects [28]. Thus, late‐life overweight and obesity may reflect survivors with protective characteristics against vascular risk factors and vascular dementia. Another hypothesis is that overweight or obese older people may have a better intake of trace elements, vitamins, and other nutrients that improve the functioning of the molecular pathways involved in the regulation of cognitive abilities [28]. A further intrinsic mechanism could be through leptin, a hormone mainly secreted by adipose tissue, that has been shown to improve learning and memory performance in animal models by regulating the synaptic plasticity of the hippocampus and the beta-process of amyloid [29], and whose high levels were associated with lower risk of dementia [30].

A clinical aspect that can mediate the possible link between body weight and cognitive state is the presence of sarcopenia [16]. Sarcopenia has been widely reported to be associated with poor functional performance in older people [31] and has been shown to be probably associated with cognitive impairment [32]. In the population covered by this study, we have not observed a high prevalence of sarcopenia, and this can be probably explained by the fact that most of the nonagenarians in our population continued to live at home, alone or with their family [10]. As already reported in a previous article from the same project, the Mugello study included a large cohort of nonagenarians living in small villages where the social community continues to support older people not to be isolated and not to live in clinics [10]. This may have influenced positively the low prevalence of sarcopenia and helps adding another hypothesis on the relationship between body weight and cognitive function since BMI, in non-sarcopenic older people, may represent a strong predictor of skeletal muscle mass that seems positively associated with improved cognitive ability [33].

The present study has several strengths and some limitations that deserve comments. The Mugello study offered many advantages to investigate the association between BMI, functional and cognitive status in older people through objective measurements of anthropometric parameters and validated tests of functioning and cognitive capacity. Indeed, only a few studies have explored the relationship between BMI and cognitive status in the older healthy population and even rarer are the data referring to the relationship between BMI, functional and cognitive status in nonagenarian groups. However, this analysis come from a cross-sectional survey that precludes the possibility of establishing causal relationships. Since we do not know the past weight of the participants in middle age, we cannot therefore evaluate the trajectory of obesity in our analysis and assess how long- or short-term obesity has affected the functional and cognitive state in old age. In addition, the population of our study that reported a normal body weight included not only those who had always been thin, but also those who lost weight unintentionally due to diseases related to the general decline in health and the development of cognitive impairment. In older people low BMI is usually a consequence of malnutrition, dysphagia, and masticatory dysfunction or bowel disorders [28, 34].

In conclusion, this cross-sectional survey from the Mugello study showed that a higher BMI is associated with lower functional capability but with better cognitive ability, supporting the hypothesis that obesity may be a protective factor against cognitive impairment in nonagenarians according to “obesity paradox”. Evidence of this paradox confirms that the risk relationship between BMI and cognitive status can change over the course of life from mid-life to late life, suggesting that optimal BMI goals in older people have yet to be established. Further studies are needed to better understand the general health status of nonagenarians and to identify strategies for its improvement.
